# Acidic retinoids in small amounts promote retinyl ester formation in neonatal lung, with transient increases in retinoid homeostatic gene expression

**DOI:** 10.1186/1743-7075-10-72

**Published:** 2013-12-19

**Authors:** Lili Wu, Reza Zolfaghari, A Catharine Ross

**Affiliations:** 1 Department of Nutritional Sciences, 110 Chandlee Laboratory, Pennsylvania State University, 16802 University Park, PA, USA; 2 Department of Nutritional Sciences, Pennsylvania State University, 16802 University Park, PA, USA

**Keywords:** Vitamin A supplementation, Acidic retinoids, Neonates, Lung

## Abstract

**Background:**

Mixing a small proportion, 10%, of retinoic acid (RA) into an oral dose of vitamin A (VA) has been shown to markedly increase retinol uptake and retinyl ester (RE) formation in the neonatal lung, as compared to VA given alone. Concomitantly, several retinoid homeostatic genes, lecithin:retinol acyltransferase (LRAT), RA-4-hydroxylase (CYP26B1), and stimulated by retinoic acid gene-6 (STRA6) were upregulated. However, whether multiple doses may act accumulatively and whether less than 10% RA can be used has not been determined.

**Methods:**

Neonatal rats were treated once on postnatal day (PD) 4 or PD14 with VA alone or VA combined with 10% RA (VARA10%) or a stable analog, Am580 (VAAm10%), or they were treated with multiple doses on PD4, 7, 11, and 14.

**Results:**

RE increased cumulatively with multiple dosing. However, LRAT, CYP26B1 and STRA6 mRNA levels were similar for single and multiple treatments, indicating a transient noncumulative impact on gene expression. Lung RE was elevated with as little as 0.5% RA (*P* < 0.05) in a single dosing study. Whereas all concentrations of VARA elevated lung RE in single dosing studies, only 10% RA increased lung RE after multiple dosing, suggesting an attenuation of RA action with repeated dosing. In contrast, VAAm10%, 2%, and 1% all significantly increased lung RE after multiple doses (*P* < 0.05), while also increasing the expression of LRAT and CYP26B1.

**Conclusions:**

These results indicate that the neonatal lung is very sensitive to acidic retinoid exposure and suggest that a VA combined with a very small fraction of acidic retinoid could be effective in increasing the lung’s storage pool of VA.

## Introduction

Retinoids have been implicated in lung development through promotion of alveolar septation, angiogenesis, and surfactant synthesis [1]. Before birth, the fetus is exclusively dependent on the maternal supply of VA. Premature infants, often with very low body weight, are at increased risk for vitamin A (VA) deficiency, probably due to a shortened period of transplacental VA supply before birth [2-5]. This population often also has a high risk for development of various pulmonary diseases [6,7]. Animal and human studies have shown that VA supplementation of lactating mothers can improve the VA status of their postnatal offspring [8-10]. Direct treatment of the offspring by oral VA supplementation also increases lung retinyl ester (RE) levels [11,12]. In several clinical trials, direct VA supplementation to newborns modestly improved VA status and reduced risk of lung injury and dysfunction [13-18]. Additionally, retinoic acid (RA), the active metabolite of VA, has also shown promising effects in lung repair by regulating genes involved in lung functioning, cell proliferation, differentiation, cell-cell communication, and cell-matrix interactions [19]. It seems likely that the effects of VA on lung development, differentiation and maintenance are the result of RA-induced regulation of many genes related to lung development, including those involved in pattern formation, matrix production, and synthesis of certain growth factors [20]. RA has been shown to increase the level of elastin mRNA in lung fibroblasts [21], and treatment of neonatal rats with RA resulted in a partial recovery of the septation process and increased formation of alveoli, even under conditions of stress [22,23].

Previously, we have reported that by combining a portion of acidic retinoid into the VA supplement, referred to as VARA, the uptake of an oral supplement of retinol and the formation of RE in the lung were enhanced approximately 5-fold as compared to the effect of VA supplementation alone [11,24], concomitant with upregulation of the levels of transcripts of retinoid homeostatic genes, including lecithin:retinol acyltransferase (LRAT) and Stimulated by RA-6 (STRA6) in short term (6 h) experiments [12]. Thus, we concluded that the redirection of VA flow into the lung by the VARA treatment, compared with VA alone, could be a result of the rapid upregulation of LRAT and STRA6 genes. Other investigators have demonstrated that LRAT and STRA6 cooperate in mediating retinol uptake and storage, for when both genes were co-expressed in retinal pigment epithelial cells the uptake and esterification of retinol exceeded that in cells expressing either one of these genes alone [25].

Our previous studies in neonatal rats or mice tested only VARA with 10 mol% of RA, referred to herein as VARA10%, which increased the content of lung RE increased ~5-fold more as compared to same amount of VA alone [11,24], and VA combined with Am580 (VAAm10%), a C-4 methyl substituted analog of all-*trans*-RA that is resistant to rapid catabolism by enzymes of the cytochrome P450 CYP26 family. LRAT and STRA6 mRNA levels were increased even more strongly as compared to VARA10%; moreover, Am580 also induced a more persistent rise in the retinoid catabolic genes, CYP26B1 in lung and CYP26A1 in liver [11,12]. However, our previous studies did not test VARA prepared with lower amounts of RA. Because minimizing unnecessary exposure to VA or RA may be an important consideration for translation of rodent studies into clinical trials, it is important to better understand the effects of dosing and acidic retinoid content on lung RE formation and retinoid homeostatic genes. Therefore the present studies were designed to answer the following questions: Do multiple treatments of VA combined with 10% acidic retinoid increase RE in the neonatal lung in an accumulative manner? Are VARA or VAAm prepared with a lower proportion of RA or Am580 still effective in promoting RE storage in the neonatal lung?

## Materials and methods

### Materials and dose preparation

VA in the form of *all-trans*-retinyl palmitate and *all-trans*-RA were purchased from Sigma-Aldrich (St. Louis, MO). Am580 was from by H. Kagechika, University of Tokyo. Either RA or Am580 was admixed with VA at a molar ratio of 10 VA to 1 RA or Am580, in canola oil (see [11,12] for rationale); oil was also used as a placebo control for oral dosing. In designated experiments the proportion of RA or Am580 was modified; each dose of VARA or VAAm delivered the same amount of VA but RA or Am580 was admixed at 10%, 5%, 2%, or 1% (VARA and VAAm), or 0.5% (VARA only), relative to VA.

### Animals and experimental designs

Animal procedures were approved by the Institutional Animal Use and Care Committee of The Pennsylvania State University. We conducted several experiments to investigate the effects of dosing schedule: 1) single early dosing on PD4; 2) single later dosing on PD14, and 3) multiple dosing throughout the period of lung septation (one dose given daily on PD4, 7, 11, and 14). Neonatal rats were randomly divided into 6 groups (*n* = 4-6/group) that received: oil (control), VA alone, RA alone, Am580 alone, VA combined with RA (VARA), or VA combined with Am580 (VAAm). Other studies tested the effect different levels of RA or Am580 in the VARA or VAAm dose, using either single or multiple dosing schedules as above. The number of animals per treatment is given in the legends to figures.

In all studies, neonatal pups were delivered by Sprague-Dawley dams fed a nutritionally complete VA adequate diet (AIN-93G) [26] containing 4 μg retinol/g. Since the pups in each study were obtained from several litters, the sexes were distributed evenly among the groups and the average body weight of each group was similar. Before dosing pups were weighed and the dose volume was adjusted to 0.4 μL/g body weight. This procedure delivered 6 mg/kg VA, which was kept constant, and the designated proportion of admixed RA or Am580. Pups were killed with prolonged carbon dioxide inhalation and exanguination 6 h after the single dose or 6 h after the final dose in the multiple dosing experiments. The lungs were removed, trimmed and weighed. All samples were frozen in liquid nitrogen immediately and then stored at -80°C for later analysis.

### Retinoid analysis

Tissues were analyzed for total retinol after saponification. Extraction, saponification, and HPLC analysis were conducted as previously described [11]. In lung tissue, >90% of total retinol is esterified [11] and thus total retinol represent RE in our studies. Retinal was not detected.

### Gene mRNA determination

Total RNA from lung tissues of individual pups was extracted using Trizol (Invitrogen, Carlsbad, CA) and then reverse transcribed using Moloney murine leukemia virus reverse transcriptase (Promega, Madison, WI). The diluted reaction product (1/20) was used for real-time PCR (rt-PCR) analysis using 2× Real Time SYBR Green PCR Master Mix (BioRad, Hercules, CA) in a final volume of 25 μl. The PCR cycling program was first set at 94°C for 3 min to activate the Taq polymerase and then at 40 cycles of 94°C for 20 s, 60°C for 30 s, 72°C for 30 s and 75°C for 1 sec using the DNA Engine2 Opticon with Continuous Florescence Detector (BioRad). The primers used to detect specific RNAs were: for rat LRAT (AF255060), 5′- CGGACCCATTTTACCCACTA-3′ (forward) and 5′- CAGACTGCAGGAAGGGTCAT-3′ (reverse); for rat CYP26B1 (NM_181087), 5′-TTGAGGGCTTGGAGTTGG T-3′ (forward) and 5′-AACGTTGCCATACTTCTCGC-3′ (reverse); for rat STRA6 (NM_0010029924), 5′-CCGATCCTGGACAGTTCCTA -3′ (forward) and 5′-CCACCTGGTAAGTGGCTGTT -3′ (reverse), for rat 18S ribosomal RNA (FQ225624), 5′- CGCGGTTCTATTTTGTTGGT-3′ (forward) and 5′- AGTCGGCATCGTTTATGGTC-3′ (forward). The mRNA expression level of each sample was corrected by calculating mRNA-to-ribosomal 18S RNA ratio. Data were normalized to the average value for the control group, set at 1.0, prior to statistical analysis.

### Statistical analysis

Data are shown as the means ± SEM. Differences between groups were tested by one-factor ANOVA followed by Fisher’s least significant difference test, using GraphPad Prism (San Diego, CA). For comparisons of mRNAs, the mean value of the control group was set to a value of 1.0, and the mean values for the other groups were converted accordingly prior to statistical testing. If group variances were unequal, the individual values were transformed to log10 values before statistical analysis. Differences with *P* < 0.05 were considered statistically significant.

## Results

### Multiple dosing resulted in a higher, accumulative increase in lung RE contents

**Figure 1:**
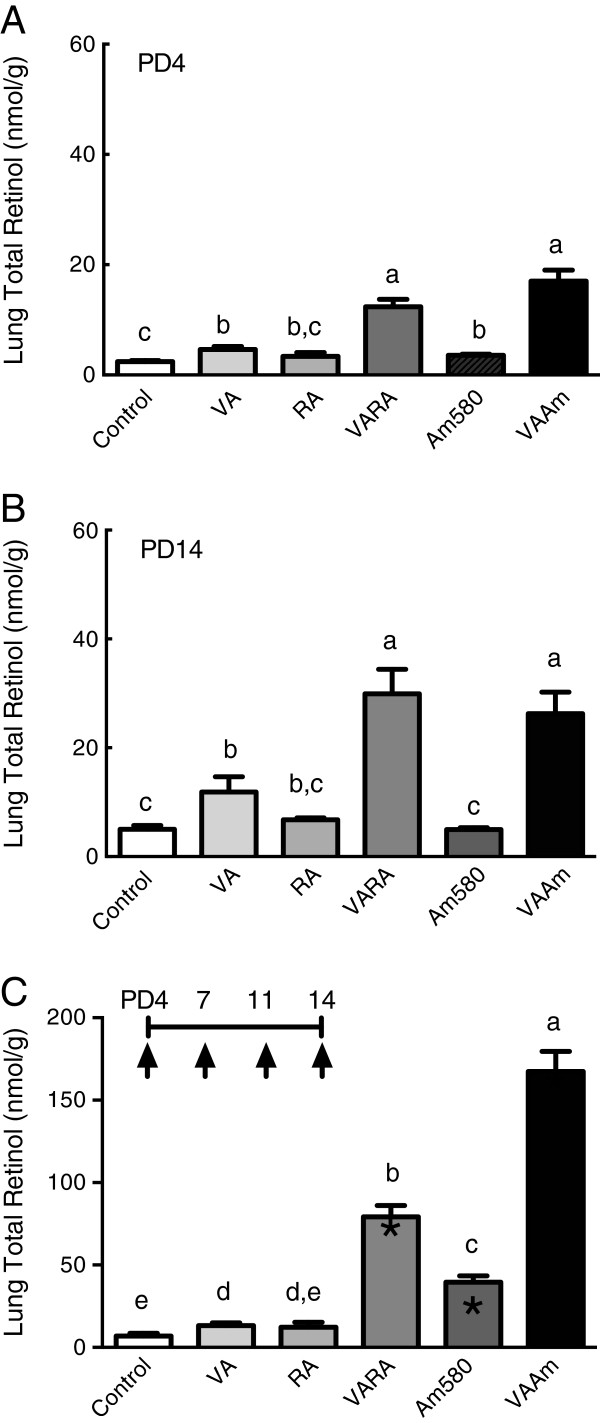
**Lung RE concentration after a single treatment or multiple treatments with VA alone, all-*trans*-retinoic acid (RA) alone, or combination of VA and 10% RA (VARA), Am580 alone, and combination of VA and 10% Am580 (VAAm).** Neonatal rats were treated with a single dose, prepared and delivered as in Experimental Methods, which was given either early during the period of lung septation (PD4) **(A)**, or near the end of the period of lung septation (PD14) **(B)**, or as multiple doses on PD 4, 7, 11, and 14 **(C)**. Lung total retinol concentration, which is mainly RE (see Materials and methods), was determined on tissue collected 6 h after the single dose or final treatment in the multiple dosing design. Data are presented as means ± SEM. Each treatment group included *n* = 4-6 neonates. Statistical analysis was conducted on log_10_-transformed data using one-factor ANOVA and Fisher’s Least Significant Difference posthoc test. Groups with different letters differed significantly, *P* < 0.05. Asterisks in panel **C** mark significant differences between the PD14 single dosing study **(B)** and multiple dosing study **(C)**, tested for each treatment by unpaired *t*-test.

Three experiments were conducted in which the timing of the dosing differed between PD4 and PD14, considered the beginning and late in the period of lung septation. Treatments were given once either on PD4 or PD14, or as repeated doses on PD4, 7, 11, and 14, with tissue collection 6 h after the single dose or final dose in the repeated dosing study. In each of these studies the treatments included oil (control), VA alone, RA alone (matched to the amount in VARA10%), VARA10%, Am580 alone (matched to the amount in VAAm10%), and VAAm10%. After single dosing on PD4 (Figure 1A), treatments with VARA10% and VAAm10% increased lung RE levels more than VA alone, which itself increased lung RE 2-fold above the control value (*P* < 0.05). These results agree with those of our previous observations that VARA and VAAm580 containing 10% of acidic retinoid synergistically increased lung RE content as early as 6 h after dosing in young rat pups [11,12]. After single dosing on PD14 (Figure 1B), a similar pattern was observed among the treatments, which suggests a consistent mechanism of response to VARA throughout the period of lung septation. When each treatment was compared between the two ages, lung RE was significantly greater on PD14 than PD4 (all *P* < 0.05 except for *P* = 0.056 between VA groups on PD4 versus PD14); therefore older neonates had both a slightly higher baseline (control) RE concentration and they responded slightly more robustly to treatment. In a third experiment, we tested multiple dosing with VARA10% and VAAm10%, given on PD4, 7, 11, and 14. Multiple dosing increased lung RE (Figure 1C) more than single dosing on PD14 (all *P* < 0.05 versus single-dose on PD14). However, for VA and RA given alone, there was no additive increase. Notably, repeated treatment with Am580 alone increased lung RE in a cumulative manner, both as compared to the control group, the VA group, and the group treated with a single dose of Am580 (all *P* < 0.05, Figure 1C). This suggests that Am580, as a stable analog of RA, redirected endogenous retinol that was present in the absence of VA supplementation into the lung.

### Multiple treatments of VARA had no cumulative effect on the expression of lung homeostatic genes, but VAAm prolonged gene expression

**Figure 2:**
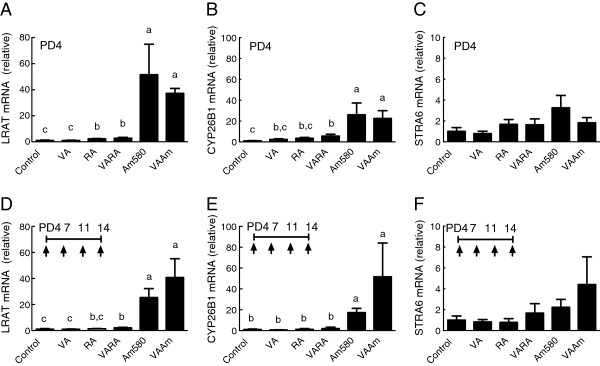
**Expression levels of LRAT, and CYP26B1 and STRA6 mRNA after treatments with single (PD4) and multiple doses of VA and acidic retinoids.** Neonatal rats received an early single dose on PD4 **(A-C)**, or multiple doses on PD4, 7, 11, and 14 **(D-F)**, as described for Figure 1A and C. Quantitative PCR results were normalized to ribosomal 18S RNA and the average value for the control group was set to 1.0 for each experiment. Data are presented as means ± SEM. Each treatment group included *n* = 4-6 neonates. Statistical analysis was conducted on log_10_-transformed data using one-factor ANOVA and Fisher’s Least Significant Difference posthoc test. Groups with different letters differed significantly, *P* < 0.05.

Next we examined single dosing versus multiple dosing with VA, RA, VARA, and VAAm on the expression of retinoid homeostatic genes. After single dosing on PD4, LRAT mRNA was significantly increased in all groups that had received a treatment containing an acidic retinoid: control ≈ VA, < RA ≈ VARA, < Am580 ≈ VAAm (Figure 2A). A similar pattern was observed for CYP26B1 although the increase was significant only for VARA, Am580, and VAAm (Figure 2B). STRA6 mRNA increased to a lesser extent and was more variable and therefore was not significantly regulated by these treatments (Figure 2C). Results for single dosing on PD14 (data not shown) were similar to those for PD4. In the multiple-dosing study, VARA significantly increased the level of LRAT (Figure 2D) but not CYP26B1 (Figure 2E) mRNA (*P* < 0.05), while both Am580 and VAAm dramatically increased LRAT and CYP26B1 mRNAs. For STRA6, results on PD14 (Figure 2F) were similar to those on PD4. Since all of these measurements were made 6 h after the single dose or the last dose in the multiple dosing protocol, the results thus suggest that RA, probably due to its very short half-life or due to its induction of CYP26B1, produced only a transient stimulation of the expression of these retinoid-homeostatic genes, while, in contrast, the stable retinoid analog Am580 induced a sustained elevation of LRAT and CYP26B1 gene expression.

**Figure 3:**
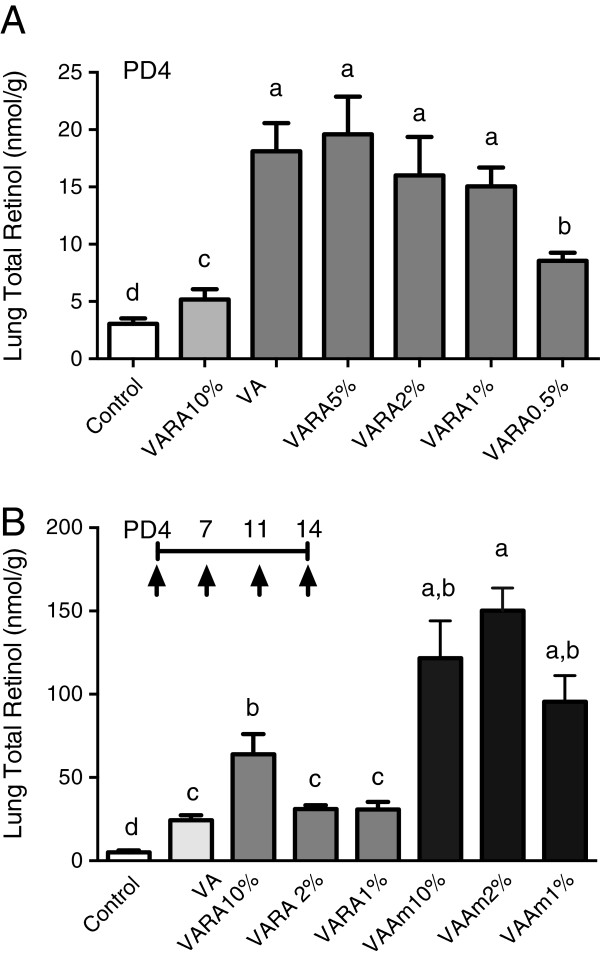
**Lung RE concentration after a single treatment with VA alone, or VARA or VAAm prepared with different percentages of acidic retinoid.** In **A**, neonatal rats were treated on PD4-5 with a single dose and tissue was collected 6 h later **(A)**. In **B**, neonatal rats were treated with multiple doses on PD4, 7, 11, and 14 and lung tissue was collected 6 h after the last dose. Data are presented as means ± SEM. Statistical analysis was conducted on log_10_-transformed data using one-factor ANOVA and Fisher’s Least Significant Difference posthoc test. Groups with different letters differed significantly, *P* < 0.05.

Previously, the lowest concentration of RA or Am580 admixed with VA that may enhance lung RE formation has not been determined. Therefore we next conducted a single-dosing experiment in which neonatal rats were treated at early neonatal age (PD4-5) with oil, VA alone, or dilutions of VARA with RA at 10%, 5%, 2%, 1% or 0.5% relative to VA. Figure 3A shows that lung RE increased after treatments with VARA10%, 5%, 2% and 1%, while VARA0.5% produced less of an increase, although still above that with VA alone (all *P* < 0.05 versus VA).

We then investigated multiple doses of VARA at dilutions of 10%, 2% and 1% RA and of VAAm at 10%, 2% and 1% Am580, each in comparison to control and VA treated pups. In this study, all doses that had been used in the single dose study (shown in Figure 1), produced a greater increase in lung RE after multiple dosing (Figure 3B), which is consistent with the cumulative effect of multiple doses shown above for VARA10%. However, VARA2% and VARA1% did not produce an increase greater than VA alone after multiple dosing. This may suggest an attenuation of the effect of RA at lower doses, due to rapid catabolism. Consistent with this notion, multiple dosing using VA combined with the stable retinoid Am580, from VAAm10%, to VAAm1%, all resulted in increased lung RE, in some cases exceeding 100 nmol/g.

### Retinoid-regulated gene expression with diluted doses of VARA and VAAm

**Figure 4:**
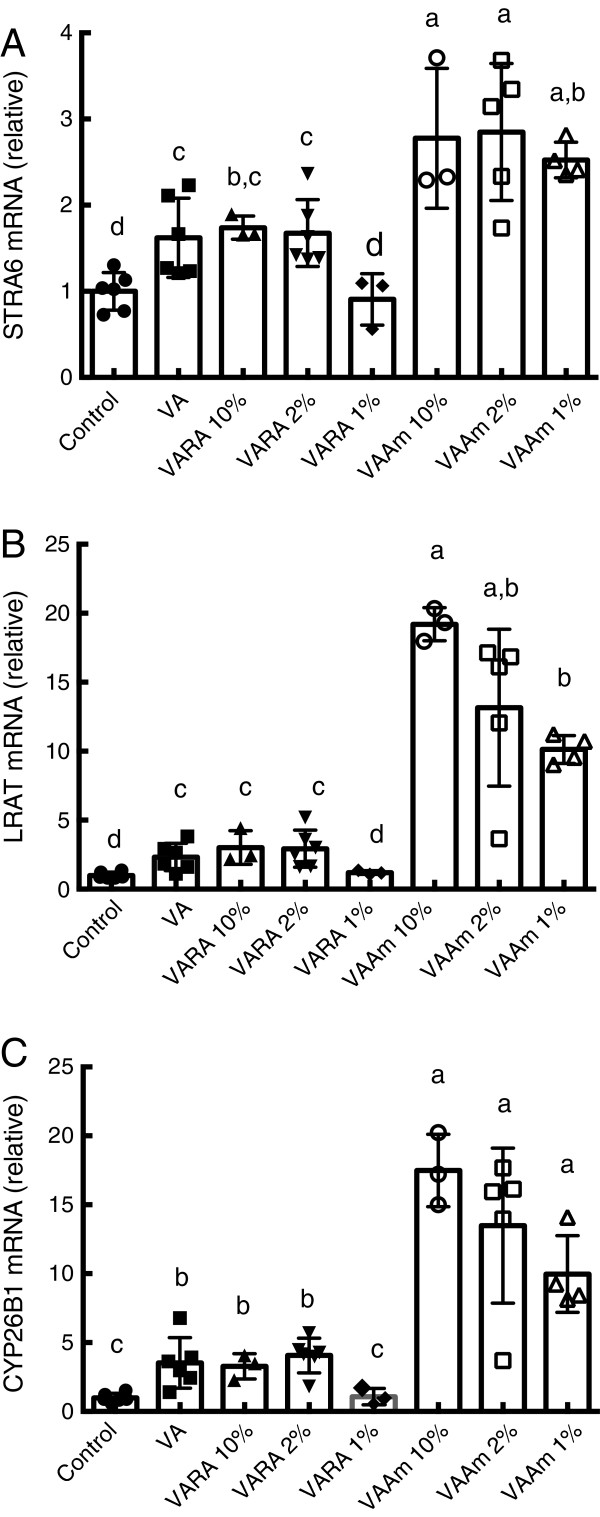
**Expression levels of STRA6, LRAT, and CYP26B1 mRNA after multiple doses of VA and VA combined with different mole percentages of acidic retinoids.** Neonatal rats were treated with on muliple doses on PD4, 7, 11, and 14. An equal amount of VA, equivalent to 6 mg retinol/kg was given either alone or combined with varying proportions of RA or Am580, from 10% to 1% compared to VA. Gene expression levels were determined for STRA6 **(A)**, LRAT **(B)**, and CYP26B1 **(C)** 6 h after the final dose. Quantitative PCR results were normalized to ribosomal 18S RNA and the average value for the control group was set to 1.0 for each experiment. Data are presented as means ± EM with values for individual neonates shown as symbols. Statistical analysis was conducted on log_10_-transformed data using one-factor ANOVA and Fisher’s Least Significant Difference posthoc test. Groups with different letters differed significantly, *P* < 0.05.

Based on these results we hypothesized that an upregulation of retinol uptake and esterification might be transient in the lung of pups treated with more diluted forms of VARA, due to rapid catabolism of the more limited amount of RA, whereas the effect of Am580 would be more persistent and thus we hypothesized that VAAm prepared with a lower proportion of Am580 would still be effective. Thus, we compared STRA6, LRAT, and CYP26B1 expression in neonatal lung using samples collected 6 h after the final dose of a study with multiple-dose treatments of either VARA at 10%, 2% and 1% of RA, or VAAm at 10%, 2% and 1% of Am580, or VA alone (as in Figure 3B). For each of these genes, the expression level after multiple treatments with VARA 10%, 2% or 1% did not differ from treatment with VA alone, while VA alone elevated expression slightly but significantly compared to the placebo-treated control group (Figure 4). By contrast, treatment with VAAm10%, 2% or 1% all significant increased the expression of STRA6 (Figure 4A), LRAT (Figure 4B), and CYP26B1 (Figure 4C), as compared to VA alone. The relative increases for LRAT and CYP26B1 were greater than those for STRA6 and exhibited a dose-response trend.

## Discussion

VA is essential in lung development and thus it is possible that supplementation with VARA during early postnatal life could be advantageous in neonates at risk of low VA status as a means of providing an adequate supply of VA to lung tissue for the production of endogenous retinoids. Previously, we reported that VARA, compared to VA alone, increased lung RE about 5 times more [12]. Biesalski [27] has referred to this synergy of VA and RA as “metabolic priming”, and we believe this is an apt description of how the RA component of the VARA preparation is working to augment VA uptake and storage in the lung. Previous studies showed that VARA was effective in increasing lung RE even in the face of inflammation [28], and VARA also has improved outcome, as suggested by reduced markers of inflammation and improved morphology in a model of hyperoxic lung injury in the neonatal mouse [29,30].

Previous studies had not tested whether repeated treatments (multiple dosing) during the neonatal period would have an additive effect on either RE formation or the level of gene expression, or whether the amount of acidic retinoid could be reduced below 10%, relative to VA, and still promote RE formation. The ability to deliver a lower percentage of RA or Am580, if effective in increasing lung RE, is important to test as, if effective, it would allow for a reduced total exposure to these compounds during early postnatal development. Our present experiments show, firstly, that single treatments on PD4 compared to PD14 resulted in very similar responses (Figure 1A and 1B), suggesting that the lung remains responsive to VARA at times near both the beginning and the end of the septation period. Secondly, multiple treatments increased lung RE contents in a manner that was both synergistic between VA and RA or Am580 and cumulative in quantity (Figure 1C vs. A and B). The cumulative increase is of interest because it suggests that intermittent, periodic dosing is sufficient to maintain an elevated level of RE in lung tissue over a period of time, especially if the acidic retinoid is persistent, and may suggest that lower doses would be effective in maintaining a moderate increase in lung RE over time. Thirdly, VARA and VAAm, each composed with 10% of acidic retinoid, did not differ from each other with respect to lung RE formation after a single dose, but VAAm produced a stronger effect than VARA after multiple dosing (Figure 1C), probably due to the longer half-life of Am580 compared to RA. Indeed, with VAAm, it appears the total dose of both retinoids combined can be reduced by half or more and still have an equivalent effect as VARA10% (Figure 3). The finding that treatment with Am580 alone, in the absence of supplementation with VA, significantly increased lung RE (Figure 1C) indicates the power of Am580 to redirect endogenous VA, presumably derived from liver or other storage tissues, into the lung. Fourthly, the results of gene expression analysis provide a possible explanation for the difference between VARA and VAAm treatments, in that treatments containing Am580 increased LRAT expression substantially, in both the single dose and multiple dosing experiments (Figure 2). The persistence of LRAT gene expression even 24 h after the final dose in the multiple-dosing study may have led to an exaggerated formation of lung RE.

The results of our dose-dilution studies with a fixed amount of VA combined with different amounts of RA and Am580 showed that small amounts of acidic retinoid significantly increased lung RE accumulation. The lowest effective concentration of RA was 0.5% in PD 4-5 neonates after a single dose (Figure 3A). Interestingly, in older PD14 neonates after multiple doses (Figure 3B), only VARA10% significantly elevated lung RE, compared either to VA alone, VARA2%, or VARA1%. In our previous 12 h experiments [12], we suggested that the activity of RA is only transient, consistent with a high turnover rate of RA in vivo [31], due to transient upregulation of retinoid homeostatic genes. We speculate that the loss of synergy observed with VARA2% and 1% in our multiple-dosing study (Figure 3B) could be due to a more rapid elimination of the small amount of RA in the VARA dose. This idea was supported by our multiple-dosing experiment using the more stable analogue of RA, Am580 (Figure 1C), in which VAAm1% increased lung RE formation compared to VA alone (Figure 3B).

Although the up-regulation of STRA6 in these studies was modest as compared to that of LRAT and CYP26 in response to either VARA or VAAM doses, it was still statistically significant (Figure 4A). The essentiality of STRA6 for retinol uptake from plasma RBP, except in the eye, has been questioned recently based on studies in STRA6 knock-out mice [32], but STRA6 might still participate in the enhanced uptake of retinol under conditions when the gene is expressed and responsive to acidic retinoids, as in lung of wildtype neonates. Studies in LRAT knock-out mice have demonstrated its importance in retinol uptake, including in adult tissues, including lung [33]. The ability of RA to upregulate these genes, even transiently, could be applied in therapies for neonates, in which VA stores are low at birth, to rapidly increase the tissue’s content of VA, which may improve the in situ formation of bioactive retinoids. Having an adequate supply of RE in lung tissue may be critical for adequate formation of bioactive retinoids. An acute accumulation and utilization of RE has been shown during the alveolar stage of lung development [34,35], when, concurrently, retinol and RA increased in lung fibroblasts, suggesting an increased demand for retinoids in normal postnatal lung development [36].

In summary, these studies have provided evidence that multiple dosing with VARA or VAAm can rapidly and markedly elevate lung RE contents in neonatal rats, and that the magnitude of increase can be controlled by the type and amount of acidic retinoid admixed with VA and the frequency of dosing. A small amount of RA admixed with VA may be a useful approach to increasing the delivery of retinol to the lungs. Our findings thus provide some clues for a more efficient and possibly better therapeutic strategy that may improve lung RE formation in neonatal lungs.

## Abbreviations

CYP26B1: Cytochrome P450 26B1; LRAT: Lecithin:retinol acyltransferase; RA: Retinoic acid; RE: Retinyl ester; STRA6: Stimulated by Retinoic acid 6; VA: Vitamin A; VARA: Supplemental vitamin A combined with retinoic acid; VAAm: Supplemental vitamin A combined with Am580.

## Competing interests

The authors declare no competing financial interests.

## Authors’ contributions

All authors have read and approved the final version. LW designed and conducted the research and prepared the draft manuscript; RZ conducted research and data analysis, and ACR planned and designed the research and prepared the final manuscript.
